# Association between Early Childhood Caries and Quality of Life: Early Childhood Oral Health Impact Scale and Pufa Index

**DOI:** 10.3390/dj7040095

**Published:** 2019-09-25

**Authors:** Ningthoujam Sharna, Mahesh Ramakrishnan, Victor Samuel, Dhanalakshmi Ravikumar, Khangembam Cheenglembi, Sukumaran Anil

**Affiliations:** 1 Dental College, Jawaharlal Nehru Institute of Medical Sciences, Imphal 795001, India; sharnapedo@gmail.com (N.S.); cheenglembik@gmail.com (K.C.); 2 Saveetha Dental College, Saveetha University, Chennai 602105, Indiadhana9677@gmail.com (D.R.); 3 Department of Pedodontics, SRM Kattankulathur Dental College, SRM University, Chennai 603203, India; 4 Department of Dentistry, Hamad Medical Corporation, P.O. Box 3350, Doha 3050, Qatar

**Keywords:** dental caries, Oral Health Impact Scale, quality of life, Early Childhood Caries

## Abstract

Early Childhood Caries (ECC) are one of the major oral diseases affecting children. ECC adversely affects the children’s as well as their parent/caregivers quality of life. The present study aims to assess the quality of life in children with Early Childhood Caries aged 6–72 months using the Early Childhood Oral Health Impact Scale. It also aims to compare the quality of life between children with pufa scores of > 0 and a pufa score = 0. A total of 238 children aged 6 months to 72 months with ECC and their parent/caregiver were included in the present study. Oral examinations of the children were performed by the principal examiner using the defs and pufa index, which was followed by a personal interview of the 13 items in the Early Childhood Oral Health Impact scale among the 238 parents/caregivers. The results showed that, overall, Early Childhood Caries have a negative impact on children’s quality of life, as assessed by the parent/caregiver. The overall Early Childhood Oral Health Impact scale score ranged from 0–32 (mean ± SD, 14.12 ± 6.72). Children with a pufa score > 0 (mean ± SD, 16.14 ± 6.27, *p* < 0.001) have significantly lower quality of life than children with pufa score = 0 (mean ± SD, 9.07 ± 4.94, *p* < 0.001). Early Childhood Caries had a negative impact on the quality of life of children aged 6–72 months. Children with a pufa score of “0” had better oral health-related quality of life than children with a pufa score > 0.

## 1. Introduction

The term Early Childhood Caries (ECC) encompasses all carious lesions occurring in the primary dentition of young children from birth to 71 months of age [1,2]. Pain and infection are the primary effects of ECC, which can cause altered eating and sleeping habits interfering with the growth of the child. This results in adverse effects on body weight and height and, ultimately, failure to thrive [3,4,5]. Dental pain as a result of untreated ECC have reported to have an adverse impact on the Quality of Life (QoL) to a degree similar to that of systemic diseases affecting the child’s anthropometric and nutritional status, socialization, lowered self-esteem, and diminished learning abilities [6]. The concept of Oral Health Related Quality of Life (OHRQoL) relates to the impact that oral health or disease has on individuals daily functioning, well-being, or quality of life [7].Until recently, the psycho-social consequences of oral conditions have received little attention.

The Early Childhood Oral Health Impact Scale (ECOHIS) was developed for assessing OHRQoL in preschool children. This instrument is comprised of four descriptive domains included in the child impact section (child symptoms domain, child function domain, child psychological domain, and child self-image/social interaction domain) and two domains for the family impact section (parent distress domain and family function domain) [8]. The pufa/PUFA index was developed by Monse et al. [9] to assess the pulpo-periapical extension of untreated caries. The uppercase PUFA are used for permanent dentition and the lowercase pufa are used for primary teeth. The PUFA/pufa index scores the presence of a visible pulp (*P*/*p*), ulceration of the oral mucosa due to root fragments (*U*/*u*), a fistula (*F*/*f*), or an abscess (*A*/*a*). Limited attempts have been made to assess the impact of untreated caries on preschool children’s quality of life with the use of pufa index. This paper presents an evaluation of the impact of ECC on QoL of children aged 6–72 months using the ECOHIS and the impact of pulpo-periapical extension of caries on the QoL of pre-school children using the pufa index, which is an area that appears to be less explored.

## 2. Materials and Methods 

### 2.1. Study Design

This was a cross-sectional study that assessed caregiver’s perception of their child’s OHRQoL in children aged 6–72 months with ECC. The study also assessed the impact of pulpo-periapical extension of caries on OHRQoL. The Scientific Review Board approved the project and the ethical approval was obtained from the Institutional Human Ethical Committee (IHEC) of Saveetha University, Chennai, India (SRB-040 30 November 2010). Informed consent was obtained from the caregiver before the commencement of the study. 

### 2.2. Sampling and Sample Size

The participants of this study were a convenience sample of primary caregivers of children who were diagnosed with ECC, in the outpatient clinic of the Department of Pediatric and Preventive Dentistry, Saveetha Dental College & Hospital, Chennai, India. Sample size was calculated based on the mean and standard deviation values of the previous study [8] using G power (Version 3.1), Type I error of 5%, and at 90% of power (1-β err prob). This gave an estimation of 218 children to be recruited. After increasing the sample size by 5% to compensate for attrition, the sample of 238 children was chosen. 

### 2.3. Inclusion and Exclusion Criteria

Children aged 6–72 months with a presence of at least one or more decayed (cavitated/non-cavitated), missing, or filled tooth surface in any primary tooth were included in the present study. Those caregivers who have been with the child since birth or at least 70% of years since birth, and caregivers who can understand the language in which the oral interview was conducted were included in the study.

Children who were medically compromised and those who declined to participate were excluded. Informed consent was obtained from the caregiver before the commencement of the study.

### 2.4. Survey Procedure

The study was undertaken in two stages comprising of intra-oral examination, which involved assessment of the caries status and pulpo-periapical diseases using the defs and pufa index [9]. This was followed by a parental oral questionnaire survey. Calibration of oral examination was carried out by three examiners on 20 children of similar age, prior to the study. The calibration was analyzed using the Intraclass Correlation Coefficient (ICC). The calculated intra examiner agreement for the pufa index was 95% and for defs was 67%.

### 2.5. Intra-Oral Examination

The examination was done in a Department of Pedodontics and Preventive Dentistry, following the WHO criteria for oral examination by the 3 calibrated and trained dentists under a dental chair light using a mouth mirror and an explorer. Prior to the examination, the teeth were not cleaned or dried. The defs index was used to score the carious tooth surfaces. It has 3 components: “d” for decayed, “e” for extracted, and ‘f” for filled surfaces. For posterior teeth, 5 surfaces were examined and recorded: facial/buccal, mesial, distal, lingual, and occlusal. For anterior teeth, 4 surfaces were examined and recorded: facial, lingual/palatal, mesial, and distal. For primary teeth, the maximum score for defs is 88 for 20 teeth.

The lowercase pufa index for primary teeth was recorded to assess the severity of pulpal diseases resulting from untreated caries. The recording of pufa index was done independently from the defs index.

***The codes and criteria for pufa index are as follows [9]:***

p/P: Pulpal involvement is recorded when the opening of the pulp chamber is visible or when the coronal structures have been destroyed by the carious process and only roots and root fragments are left. No probing was performed to diagnose pulpal involvement.

u/U: Ulceration due to trauma from sharp pieces of a tooth is recorded when sharp edges of a dislocated tooth with pulpal involvement or root fragments have caused traumatic ulceration of the surrounding soft tissues, e.g., tongue or buccal mucosa.

f/F: Fistula is scored when a pus-containing swelling related to a tooth with pulpal involvement is present.

a/A: Abscess is scored when a pus-containing swelling related to a tooth with pulpal involvement is present.

For an individual person, the score for pufa ranges from 0 to 20 for primary dentition. The sample was divided into 2 subgroups based on the pufa score. Group 1 children had a pufa score = 0 and Group 2 children had a pufa score > 0.

### 2.6. Questionnaire Survey

All caregivers of 238 children were subjected to an oral interview using a structured instrument known as the ECOHIS [8]. The items were read out along with the responses to the caregivers and they were instructed to answer the response that best describes their children’s experience or their own. 

The ECOHIS comprises of 13 items, which includes the following responses: “Never”, “Hardly ever”, “Occasionally”, “Often” and “Very often” plus a “Don’t know” option recoded as “missing”. The scale is scored using a simple Likert frequency type scale, ranging from 0–4 [10]. The item score achieved were added to create a total scale score in a range of 0–52. Higher scores were specified as presenting more problems and/or greater impact on quality of life [8].

### 2.7. Statistical Analysis

Both questionnaire and oral examination forms were manually checked for completion of data. Following the completion of the parental survey and dental examination, the participants were divided into 2 groups based on their pufa scores: Group 1 (pufa score = 0) and Group 2 (pufa score > 0). The analysis was carried out using the SPSS software (Ver. 17.0 for Windows, SPSS Inc. Chicago, IL, USA). Statistical differences between the 2 groups were assessed using the independent *t* test. Correlation between the ECOHIS score and the pufa and defs score was analyzed using Spearman’s correlation test. For all the tests, the level of statistical significance was set at 5%.

## 3. Results

### 3.1. Sample Characteristics

A total of 238 children (53.8% males and 46.2% females) and caregiver pairs consented to participate in the present study, as shown in Table 1. The mean age of the participants was 54.01 ± 13.45 months (mean ± SD). Among these participants, 170 children had a pufa score of > 0 (71.4%) while the remaining (*n* = 68, 28.6%) had no pulpo-periapical extension of caries.

### 3.2. Distribution of Responses

Table 2 shows the distribution of responses for the whole sample, wherein the most prevalent items in the child impact section were “pain” and “difficulty while eating and drinking” “Smiling” and “talking” were influenced the least in these children. Similarly, impacts related to oral health of the children were also observed in parental domains, which results in psychological and financial constraints.

For children in Group 1 (pufa score = 0), it was observed that no parent reported any impact on the child by avoiding smiling and talking with other children in the child impact section. However, items related to the family impact section, such as feeling guilty and being upset was reported more frequently, as depicted in Table 3.

The distribution of responses for ECOHIS responses for children with a pufa score > 0 showed that the four domains (symptoms, function, psychological, self-image/social interaction domain) in the child impact section were influenced more than the children with nil pufa scores. Similarly, domains related to parental distress and functions were also greatly influenced in this group.

### 3.3. Overall ECOHIS Score

The overall mean ECOHIS score observed was 14.12 ± 6.72 with an observed range of 0–32. The parent distress domain showed the highest mean value while the least mean ECOHIS score was observed for the child self-image/social interaction domain, as presented in Table 4. Overall, for the two sections, the family impact section showed a higher mean ECOHIS in comparison to the child impact section. Considering the floor effect for the total ECOHIS score as shown in Table 4, it was observed that the no impact [floor effect, i.e., the proportion with score of “0” (% score 0)] were reported by only 2.1% parents out of the 238 parents. The highest floor effect was seen in the child self-image/social interaction domain and the lowest floor effect was seen in the parental distress domain.

### 3.4. Comparison between Pufa Scores

A significant difference was observed in the ECOHIS scores between children with or without pulpo-periapical disease, as shown in Table 5. Relatively higher mean scores were observed in all the domains in children with a puf score of > 0.

### 3.5. Correlations between ECOHIS Scores and Pufa/Defs Scores

Table 6 depicts stronger correlation between the pufa scores and the ECOHIS score in comparison to the correlation of defs scores with the ECOHIS scores.

## 4. Discussion

Children are considered an integral part for dental public health research and practice [11]. However, over the past several decades, OHRQoL assessment tools have been developed mostly for adults and the elder population with a limited number of assessment tools available for children [8]. The ECOHIS is a validated proxy measure for assessing the oral health related quality of life in preschool children developed by Paheletal. [8] in the United States. This paper presents the result of our cross-sectional study, which evaluated the QoL in preschool children with ECC. The original version of the validated instrument, known as the ECOHIS developed to assess the OHRQoL in preschool children [8], was used in the present study. As emphasized by Davies [12], in developing countries, children with ECC suffer the same deprivations as people living in poor economic circumstances in developed countries. Consequently, studies in such an environment may lead to interventions, which, in the long term, may prove to be more successful. Hence, there exists a great need for studies to evaluate OHRQoL in India, which was addressed through this study.

In our study, we performed a face-to-face interview with the primary caregivers, which is similar to the mode of administration used by Scarpelliet al. [13] for validating a Brazilian ECOHIS (B-ECOHIS) version, which is contrary to the original ECOHIS [8] version where they have used the self-completed mode of administration. The mode of administration used in the present study is supported by studies, which have indicated that a face-to-face interview provides a more favorable picture of QoL assessment when compared to the self-administered mode and is more relevant in developing economies with low literacy rates [14,15,16]. To compare the QoL among the children with ECC, we have used the pufa index, which was developed by Monseet al. [9].This index is used to quantify the prevalence and severity of pulpo-periapical diseases resulting from untreated dental caries [9]. Presenting data based on the pufa index will provide health planners with relevant information, which is complementary to the dmft. However, providing decision makers only with dmft scores leaves them unaware of the high levels of untreated caries, their severity, and associated health and QoL consequences [9].

Following analysis of the distribution of responses in our study, it was observed that, in the child impact section, the most prevalent impacts were related to children’s symptoms and functions while impacts related to self-image/social interaction were the least prevalent. This finding is consistent with previous studies [8,10,17]. However, in contrast to the findings in previous studies, our mean scores were high in the family impact section for the whole scale. This data, nevertheless, was consistent with the study conducted by Locker et al. [18], which indicated that oral conditions affect parent and family activities as well.

Prior to the initiation of the study, it was decided to recode “Do Not Know’’ responses as missing, as suggested by Jokovicet al. [19].Since the present study did not have any “Do Not Know’’ response, this was not taken into account. The lacks of “Do Not Know’’ responses in this study can be attributed to the fact that majority of the respondents were mothers of children who reported only after the onset of symptoms related to caries. Results pertaining to correlations between the ECOHIS score and the pufa/defs scores showed greater association between pufa scores and ECOHIS scores. This finding suggests that pulpal symptoms have a stronger link with the underlying construct of the OHRQoL. 

One of the hypotheses of our study was that children with a pufa score of more than zero will have a poorer OHRQoL than the children with a pufa score of zero. It could be observed that children with a pufa score of more than zero had a higher negative impact on both the sections of the scale when compared to the children with a pufa score of zero. Pahelet al. [8] reported that children with elevated dmft scores had relatively poorer QoL. In the present study, the severity of ECC was assessed using the pufa index, in order to evaluate the consequences of pulpo-periapical extension of caries on the QoL of children. The dmft index emphasizes only on caries, restored and extracted tooth as a consequence of dental caries. However, it fails to present details on the clinical consequences of untreated dental caries, such as pulpal involvement and dental abscess, which may negatively influence the OHRQoL than the presence of carious lesions alone [9]. In the present study, the majority of the caregivers reported the negative impact of oral health problems on their children’s QoL, which leads to nonexistence of the floor effect (2.1%) for the whole scale. This is depicted in Table 4. The floor effect, i.e., the proportion with a score of “0” was reported by 17.9% and 5.9% parents on the child and family sections. This signifies that only a small fraction of parents reported “no impacts” on the child and family impact sections, respectively. However, a substantial floor effect was observed on the child self-image and social interaction (95.0%), child psychological domain (58.0%), and child symptom domain (26.9%) for the child impact section where, in the family impact section, evident floor effects were seen in the family function domain (23.5%). This is in sync with the findings from another study by Pekeret al. [20] where the floor effect was more evident in the child’s self-image and social interaction (43.5%), child’s symptoms (27.3%), and child’s psychological domain (21.5%) for the child impact section while, for the family impact section, the family function domain showed higher floor effects (52.9%). The floor effect for the ECOHIS value appears to be in accordance with the disease characteristics of the sample wherein the entire study sample taken was affected by ECC. 

The data for the present study was obtained from a convenience sample and, therefore, our result provides evidence for its performance in this population only. Hence, it cannot be extrapolated to the general population. Further research needs to be conducted in different locations and different patient populations to verify and confirm the extent of the findings reported in this paper. Furthermore, including children without ECC would have facilitated a further comparison.

## 5. Conclusions

Within the limitations present, it can be concluded that, with the use of the pufa index, the present study offered additional information on the significant negative influence of pulpal involvement and abscess on the OHRQoL in children and their families than the presence of caries alone. In addition, the pufa index can act as a medium for health planners for showing parents the real picture for the causes of pain and infection due to caries. It can help pediatric dentists to create awareness among the caregivers about the possible negative impacts of Early Childhood Caries in children’s quality of life and help them to prevent caries.

## Figures and Tables

**Table 1:** Demographic details of the whole sample (*N* = 238).

Variables	Frequency (*N*)	Percentage (%)
**Total**	238	100%
**Age (months)**		
6–36months	51	21.4%
37–72months	187	78.6%
**Gender**		
Male	128	53.8%
Female	110	46.2%
**Maternal Educational Qualification**		
8th grade or less	39	16.4%
High school	127	53.4%
College degree	72	30.2%
**pufa scores**		
pufa score > 0	170	71.4%
pufa score = 0	68	28.6%

**Table 2:** Distribution of ECOHIS responses for the whole sample (*N* = 238).

Impacts	Never *N* (%)	Hardly Ever, Occasionally, Often, and Very Often *N* (%)	Do Not Know *N* (%)
**Child Impacts**			
Pain	64 (26.9)	174 (73.1)	0 (0)
Drinking	113 (47.5)	125 (52.5)	0 (0)
Eating	97(40.8)	141 (59.3)	0 (0)
Pronouncing	223 (93.7)	15 (6.2)	0 (0)
Absence	165 (69.3)	73 (30.8)	0 (0)
Sleeping	146 (61.3)	92 (38.7)	0 (0)
Frustrated	180 (75.6)	58 (24.3)	0 (0)
Smiling	229 (96.2)	9 (3.7)	0 (0)
Talking	228 (95.8)	10 (4.2)	0 (0)
**Family Impact**			
Upset	27 (11.3)	211 (88.3)	0 (0)
Guilty	26 (10.9)	212 (89.1)	0 (0)
Work	87 (36.6)	151 (63.4)	0 (0)
Financial	106 (44.5)	132 (65.5)	0 (0)

**Table 3:** Distribution of Early Childhood Oral Health Impact Scale (ECOHIS) responses for the subgroups, divided based on the pufa index score-group 1 (pufa score = 0), *N* = 68, and group 2 (pufa score > 0), *N* = 170.

Impacts	Group 1 (Pufa Score = 0) *N* = 68			Group 2 (Pufa Score > 0) *N* = 170		
	Never *N* (%)	Hardly Ever, Occasionally, Often, and Very Often *N* (%)	Do Not Know *N* (%)	Never *N* (%)	Hardly Ever, Occasionally, Often, and Very Often *N* (%)	Do Not Know *N* (%)
**Child Impacts**						
Pain	35 (51.9)	33 (48.5)	0 (0)	29 (17.1)	141 (82.9)	0 (0)
Drinking	46 (67.7)	22 (32.4)	0 (0)	67 (39.4)	103 (60.7)	0 (0)
Eating	47 (69.1)	21 (30.9)	0 (0)	50 (29.4)	120 (70.6)	0 (0)
Pronouncing	67 (98.5)	1 (1.5)	0 (0)	156 (91.8)	14 (8.3)	0 (0)
Absence	52 (76.5)	16 (23.6)	0 (0)	113 (66.5)	57 (33.5)	0 (0)
Sleeping	56 (82.4)	12 (17.6)	0 (0)	90 (52.9)	80 (47)	0 (0)
Frustrated	64 (94.1)	4 (5.9)	0 (0)	116 (68.2)	54 (31.7)	0 (0)
Smiling	68 (100)	0 (0)	0 (0)	161 (94.7)	9 (5.4)	0 (0)
Talking	68 (100)	0 (0)	0 (0)	160 (94.1)	10 (6)	0 (0)
**Family Impact**						
Upset	17 (25.0)	51 (75)	0 (0)	10 (5.9)	160 (94.1)	0 (0)
Guilty	15 (22.1)	53 (77.9)	0 (0)	11 (6.5)	159 (93.6)	0 (0)
Work	29 (42.6)	39 (57.4)	0 (0)	58 (34.1)	112 (66)	0 (0)
Financial	39 (57.4)	29 (42.6)	0 (0)	67 (39.4)	103 (60)	0 (0)

**Table 4:** Descriptive statistic pertaining to ECOHIS scores.

Impacts	Number of Items	Possible Range	Observed Range	Floor Effect	Mean (SD)	Median (IQR)
**Child impact section**	9	0–36	0–24	17.9%	6.66 (4.97)	7.00 (8.0)
Child symptoms	1	0–4	0–4	26.9%	1.66 (1.19)	2.00 (2.0)
Child function	4	0–16	0–10	24.8%	3.37 (2.65)	3.50 (5.25)
Child psychological	2	0–8	0–8	58.0%	1.45 (2.00)	0.00 (3.0)
Self-image and social interaction	2	0–8	0–8	95.0%	0.19 (0.95)	0.00 (0.0)
**Family impact section**	4	0–16	0–15	5.9%	7.55 (3.45)	8.00 (4.0)
Parental distress	2	0–8	0–8	9.7%	4.70 (2.00)	5.50 (2.0)
Family function	2	0–8	0–8	23.5%	2.85 (2.34)	2.00 (3.0)
**Total score**	13	0–52	0–32	2.1%	14.12 (6.72)	14.00 (9.25)

**Table 5:** Comparative analysis of QoL between children with and without pulpo-periapical complications. (Independent *t*-test, statistically significant at *p*-value < 0.001).

Scale/Subscale	Mean ECOHIS Score (SD)		*p*-Value
	Group 1 (Pufa = 0)	Group 2 (Pufa > 0)	
**Child impact section**	3.24 (3.52)	8.34 (3.52)	<0.001
Child symptoms	0.97 (1.09)	1.93 (1.11)	<0.001
Child function	1.82 (2.16)	3.99 (2.58)	<0.001
Child psychological	0.44 (1.10)	1.85 (2.15)	<0.001
Self-image and social interaction	0.00 (0.00)	0.26 (1.12)	0.003
**Family impact section**	5.99 (3.48)	8.81 (3.23)	<0.001
Parental distress	3.74 (2.28)	5.08 (1.74)	<0.001
Family function	2.25 (2.14)	3.09 (2.37)	0.012
**Total score**	9.07 (4.94)	16.14 (6.27)	<0.001

**Table 6:** Correlation between ECOHIS scores with pufa and defs scores. [Spearman’s correlation test (ρ(rho), statistically significant at *p* < 0.001).]

Variables	n	Correlation ρ(rho)	*p*-Value
ECOHIS SCORE and pufa Index Score	238	0.431	< 0.001
ECOHIS SCORE and defs Index score	238	0. 288	< 0.001
